# Post-flight confusion: does flying affect the brain?

**DOI:** 10.1192/bji.2020.1

**Published:** 2020-08

**Authors:** Gianetta Rands, Thomas McCabe, Chris Imray

**Affiliations:** 1 Independent Psychiatrist, London, UK. Email: grands@doctors.org.uk; 2 ST4 in Old Age Psychiatry, West of Scotland Higher Psychiatry Training Scheme, UK; 3 PhD, FRCS, FRCP, FRGS, Consultant Vascular and Renal Transplant Surgeon, Director of Research and Development, University Hospitals Coventry and Warwick NHS Trust, UK

**Keywords:** Aetiology, dementia, organic syndromes, clinical neurology, cognitive neuroscience

## Abstract

This paper describes a condition termed post-flight confusion using anecdotal and clinical observations. It reviews research from the fields of aviation and altitude medicine and how this could apply to some physiological changes that happen during commercial flights. The collection of symptoms observed is similar to those of delirium. More research is needed to validate these observations, to identify the risks of flying for older people and to consider not only how to minimise these risks but whether this situation contributes to our knowledge about the aetiologies of delirium and dementias.

Flying is now a common part of modern life. In 1998, it was estimated that 1 in 10 passengers who passed through UK major airports were over the age of 65 and mostly travelling for ‘leisure’ purposes.^1^ Recent data about the ages of air passengers are difficult to find and usually summarised in statements such as ‘Senior travel is becoming a massive part of the travel industry’.^2^ Observations about post-flight confusional states have been made for over a decade^3^ and anecdotal adverse events are known to many clinicians working with older adults. These observations have been described in medical literature to the level of case reports.^3,4^ This paper describes some of these observations and considers likely aetiological factors.

## The passenger cabin environment

Most of the research about the effects of higher altitudes on human physiology has been done on pilots and crew members in good physical health. There is no published research reflecting the challenges facing the older traveller with complex comorbidities. The cabin environment is artificially controlled, except for radiation, which is monitored. Planes cruise at altitudes of 30 000–40 000 ft (Table 1) and at this altitude air pressure is around 18.6 kPa, which is incompatible with life. Currently, cabin pressures are controlled at 74.5–84.1 kPa, corresponding to 6000–8000 ft altitude (sea level is about 96.5 kPa).^3^ Some modern jets control their cabins to 6000 ft and claim that fewer symptoms of ‘jet lag’ are experienced by their passengers.

**Table 1:** Altitudes in feet and metres above sea level and known physiological changes in healthy human adults

Altitude, feet	Altitude, m	Example	Approximate peripheral oxygen saturation, %^a^	Other known physiological changes^b^
0	0	Sea level	98–100%	Baseline
6000	1829	Latest jet plane cabin pressure	About 90%	Respiratory and heart rate increase
8000	2438	Usual cabin pressures; ‘high altitude’ refers to 2500 m	85–90%	‘Altitude sickness’,^c^ a sense of euphoria, headaches
15 775	4808	Mont Blanc summit	80%	Altitude sickness is common
9843–16 404	3500–5800	‘Very high altitude zone’	≤80%	Respiratory and heart rates continue to increase
16 404–26 247	5800–8000	‘Extreme altitude zone’	<75%	As above; cerebral oedema and retinal haemorrhages can occur
>26 247	>8000	‘Death zone’	<55%	Mountaineers require supplementary oxygen
29 030	8 848	Mount Everest summit	<55%	Acclimatisation or supplementary oxygen needed
35 000–40 000	10 668–12 192	Airplane cruising altitudes	Very low	Incompatible with life

a. The rate of change in oxygen levels can affect physiological response.

b. There is individual variation in physiological response to increasing altitude and in how people feel with reduced oxygen levels.

c. Symptoms of altitude sickness include headache, nausea, vomiting, dizziness, fatigue and insomnia. Signs of altitude sickness include: raised pulse, respiratory rate, diastolic pressure and body temperature; peripheral and pulmonary oedema.^5^

Planes ascend to cruising heights in 20–30 min and descend at similar speed. Low air pressure is associated with expansion of air spaces (Boyle's law), which are present in bowels, sinuses and recent surgical sites. Lower air pressures are associated with peripheral oedema and potential bleeding from varices.

At sea level, peripheral oxygen saturation of the blood (*S*_pO2_) is normally 97–99%, whereas at 6000–8000 ft altitude there is a 20–26% reduction in available oxygen, which results in oxyhaemoglobin saturations of 83–85%. Anecdotally, using a small pulse oximeter, *S*_pO2_ values during a flight were entirely as predicted by physics, starting and ending at ground level at 98–99%, with a range of 83–92% from 20–30 min into the flight until descent at destination. A compensatory increase in pulse was sometimes noted. Although respiratory rates were not recorded, these increase as *S*_pO2_ decreases.

Humidity at cruising cabin pressures can be as low as 1–20%. Our ‘comfort zone’ is 50–65%. Low humidity can result in dehydration and reduced peripheral perfusion.

There are no internationally agreed standards for cabin air quality. Cabin air may contain elevated levels of carbon dioxide (CO_2_), ozone and microbes that would be illegal in office spaces.^3^

## Basic physiology and brain responses to hypoxia and other aspects of in-flight environments

Cerebral perfusion pressure is auto-regulated as the difference between blood pressure and intracranial pressure. Arterial carbon dioxide levels and local metabolic activity both increase cerebral perfusion. Low arterial oxygen rapidly results in increases in respiratory and heart rates and, over time, an increased haematocrit. As the skull has a fixed internal volume, it is the cerebrospinal fluid that buffers brain volume changes. Lower levels of inspired oxygen result in increases in intracranial pressure and can subsequently compromise perfusion of some brain regions.

**Fig. 1:**
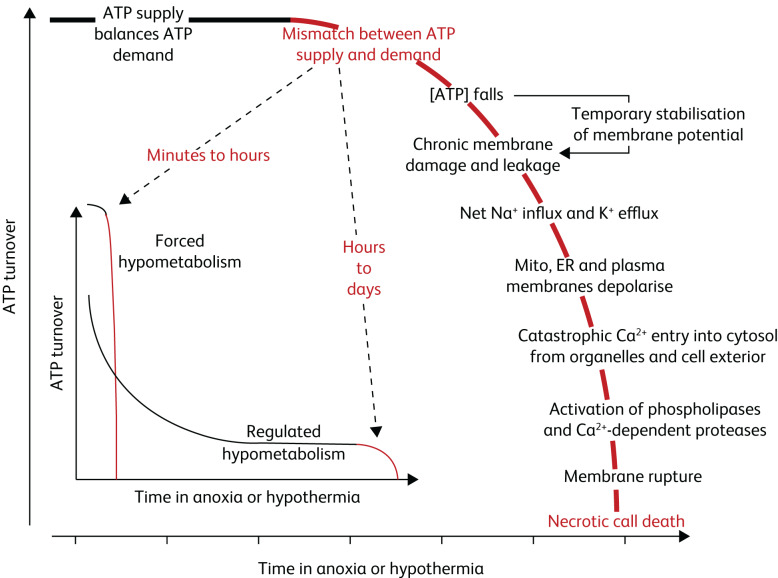
Adenosine triphosphate (ATP) turnover of cells as a function of time exposed to anoxia and hypothermia (reproduced with permission^6^). Mito, mitochondria; ER, endoplasmic reticulum.

Adenosine triphosphate (ATP) is the universal cellular energy currency, and as the levels of oxygen drop, a relative mismatch between the cellular ATP supply and demand can develop. This can result in cellular hypometabolism (Fig. 1). Depending on duration and the efficacy of the physiological response, this hypometabolism can result in cerebral cellular hypoxia and subsequent cell damage. Evidence indicates that physiological auto-regulation is impaired by increasing age, sleep, alcohol and hypnotics, and there may be other factors, such as various medications.

There are a number of potential options that could reduce this effect, such as supplementary oxygen. Environmental modifications that might be beneficial may also have adverse effects. Modest increases in cabin pressure would improve cerebral oxygen delivery to all passengers but would have costly implications for airplane design.

## Cognitive effects of high altitude

Interest in the cognitive effects of high altitude started with research by balloonists James Glaisher and Henry Coxwell in the 1860s, when both men became unconscious on rapid ascent to altitudes of approximately 25 000 ft.^7^ In 1932, these effects were demonstrated with handwriting samples that became more jumbled with increasing altitude. A number of research studies have demonstrated specific cognitive difficulties at altitudes. For instance, in 2017 Griva *et al*^8^ assessed a range of cognitive functions after ascent to Everest base camp and found that attention, learning, verbal abilities and executive function declined to variable degrees with ascent to altitude. For trekkers, ascent to altitude was clearly slower and more effortful than for passengers in jet planes. There was a wide inter-individual variability and the impairments were greater in older trekkers. It would appear that the older individual's cerebral circulation is more susceptible to relatively subtle changes in inspired oxygen levels.

## Discussion

For many years, some airlines have been aware that their passengers may suffer respiratory problems after flying. They quote the figure as 1 in 4 passengers suffering this condition.^9^ They attribute this to the usual cabin pressures, which are equivalent to an altitude of 8000 ft. Aircrafts manufactured using the newest technology have a fuselage made of carbon fibre reinforced plastic, which does not suffer ‘metal fatigue’, and hence their cabin pressures can be greater. Metal fatigue occurs in aluminium and other metals because planes expand and contract during ascent and descent owing to increased differential pressures between cabin and surrounding air. The higher cabin pressures that can be achieved with this structure of fuselage (and newer versions in production) result in lower altitude equivalent pressures of approximately 6000 ft and, it is claimed, only 1 in 12 passengers suffer subsequent respiratory distress from this acute, short-term altitude exposure.

More recently it has been suggested that jet lag could be similar to acute mountain sickness, which affects some individuals above 6500 ft altitude. Focus remains on symptoms such as headache, nausea, lack of appetite, lack of energy and sleeplessness, with acknowledgement of the disruption of diurnal rhythms,^10^ rather than on any longer-term post-flight symptoms.

Post-flight confusion could be construed as a form of delirium. Acute confusional state or delirium is a common clinical syndrome characterised by disturbed consciousness, decline in cognitive function or changes in perception. It is estimated to occur in 10–20% of medical patients admitted to hospital and is strongly associated with increased mortality, even after readjustment for severity of disease.^11^ Many causes of delirium are considered in a standard medical admission ‘work-up’. However, sometimes underlying aetiology is not found,^12^ in which case we suggest that a history of recent flying should be considered.

In-flight medical emergencies are relatively rare, although they may be underreported because of commercial interests and maintaining customer confidence. Although the common cardiovascular, respiratory and surgical complications associated with pressure changes have been addressed in guidance and regulations produced by aviation authorities and commercial airlines, there is little about cognitive symptoms that may be caused or exacerbated by the described environmental changes. Indeed, mental health-related advice for flying in general is poorly approached by aviation authorities. ‘Unpredictable, aggressive, dis-organised or disruptive’ behaviour is cited in flying guidance from the Civil Aviation Authority, which is unhelpful to the casual reader when compared with guidelines on physical health.^13^

## Conclusions

The laws of physics determine environmental changes at altitude. Research from the fields of aviation and altitude medicine informs us of human physiological changes in fit young men (as this research is rarely done on other individuals). Airlines are becoming aware that current cabin environments could be associated with symptoms similar to those found in altitude sickness. With few exceptions, such as deep vein thrombosis (DVT), there remains no systematic research into the health of passengers after they leave their destination airports.

Investigation is needed into the effects of flying environments and the manner in which human physiology adapts to high altitudes at different stages of our lifespan.

Post-flight confusion is anecdotally being seen more often and clinicians should identify people at risk and consider ways to minimise this risk. Research in this field may shed light on some mechanisms of delirium and contribute to our knowledge about aetiologies of dementia syndromes. This topic could have far-reaching effects for individuals flying and for the wider aviation business.
